# Effects of Sun Drying Versus Vacuum Freeze-Drying on the Protein Quality of *Hippocampus abdominalis*: Nutritional Composition, Physicochemical Properties, and Proteomic Profiles

**DOI:** 10.3390/foods15183236

**Published:** 2026-09-13

**Authors:** Yu Zhang, Jing Sun, Han Hu, Lin Zhang, Xuan Ma, Jiangying Heng, Xiaofeng Xue, Rui Chen, Feng Wang

**Affiliations:** 1 College of Biochemical Engineering, Beijing Union University, Beijing 100101, China; 2 State Key Laboratory of Resource Insects, Institute of Apiculture Research, Chinese Academy of Agricultural Sciences, Beijing 100193, China; 3 Innovation Center of Pesticide Research, College of Science, China Agricultural University, Beijing 100193, China

**Keywords:** *Hippocampus abdominalis*, sun drying, vacuum freeze-drying, amino acids, proteomics

## Abstract

Drying is an important processing method for extending the shelf life of aquatic products; however, different drying methods may exert distinct effects on protein quality. At present, the effects of sun drying and vacuum freeze-drying on the protein quality of *Hippocampus abdominalis* remain unclear. This study compared the protein content, amino acid composition, and physicochemical properties of *Hippocampus abdominalis* subjected to the two drying methods and further characterized changes in the proteomic profile using proteomic analysis. The results showed that the sun-dried group had a higher crude protein content (68.57% vs. 61.86%), whereas the freeze-dried group exhibited greater total amino acid retention (58.79% vs. 48.87%), water-holding capacity (WHC), and oil-holding capacity (OHC). However, no significant differences were observed in protein solubility or surface hydrophobicity between the two groups. Proteomic analysis further revealed that most differentially abundant proteins showed higher relative abundance in the freeze-dried group and were mainly associated with muscle structural stability, amino acid metabolism, and energy metabolism. Overall, the two drying methods affected the protein quality of *Hippocampus abdominalis* at multiple levels, including nutritional composition and physicochemical properties, while proteomic analysis provided further molecular-level insights into these quality differences.

## 1. Introduction

Global aquaculture is increasingly shifting toward high-value, functional marine biological resources, among which seahorses (*Hippocampus* spp.) have attracted considerable attention owing to their distinctive nutritional properties and traditional medicinal value. Among cultured seahorse species, *Hippocampus abdominalis* stands out for its rapid growth, high thermal tolerance, and well-established large-scale captive breeding techniques, making it a commercially promising candidate for temperate aquaculture systems. Between 2004 and 2018, a total of 7147 *Hippocampus abdominalis* individuals were recorded as international imports, while 27,785 individuals were recorded as exports. Notably, captive-bred individuals accounted for approximately 94.4% of the recorded imports and 96.2% of the recorded exports, indicating that captive breeding played a dominant role in the international trade of this species [1]. From a nutritional perspective, the muscle tissue of this species is characterized by a high protein content and a low lipid level, making it a promising source of high-quality marine protein [2]. However, its high protein content and high water activity also make it highly susceptible to post-harvest spoilage. Therefore, effective dehydration to reduce moisture content is essential for preserving the nutritional integrity of cultured seahorses and extending their commercial shelf life [3].

Drying is widely used for the processing and long-term preservation of aquatic products. By reducing moisture content and water activity, drying can inhibit microbial growth and stunt deterioration reactions such as lipid oxidation and enzymatic browning, thereby extending product shelf life [4]. Sun drying is one of the traditional methods commonly used for drying aquatic products because of its simple operation, low equipment requirements, and low cost. However, the drying process is highly dependent on environmental conditions, and fluctuations in temperature, humidity, and solar radiation may affect both the drying rate and the quality of the final product [5]. Prolonged exposure to air and light may also increase the risk of protein oxidation, degradation, and structural alterations. In contrast, vacuum freeze-drying achieves dehydration through pre-freezing, followed by sublimation of ice crystals under vacuum, thereby avoiding prolonged exposure to high temperatures. Consequently, it has been widely used for the drying and preservation of thermally sensitive foods; however, its relatively long drying cycle, high energy consumption, and elevated processing costs limit its large-scale industrial application to some extent [6]. Notably, ice crystal formation, freeze concentration, and interactions at the ice–water interface during freezing may also induce protein oxidation and structural alterations, indicating that freeze-drying is not a completely non-destructive dehydration method [7]. Therefore, sun drying and freeze-drying create markedly different conditions in terms of temperature, moisture migration, and oxygen exposure, which may affect the proteins of *Hippocampus abdominalis* to different extents. A systematic comparison of the protein nutritional composition, physicochemical properties, and proteomic profiles of *Hippocampus abdominalis* subjected to these two drying methods may provide further insight into the relationship between drying conditions and changes in seahorse protein quality.

The effects of drying methods on the protein quality of aquatic products have been extensively investigated across various species internationally. For example, studies on squid (*Todarodes pacificus*) fillets have demonstrated that freeze-drying effectively retains myosin integrity and results in an amino acid profile similar to that of raw samples, whereas hot-air drying causes severe damage to myosin secondary and tertiary structures [8]. Similarly, research on golden pompano (*Trachinotus ovatus*) revealed that freeze-drying induces less tissue damage and preserves more intact myofibrils compared to hot-air drying and heat pump drying [9]. Despite this wealth of international research on other aquatic species, the specific effects of sun drying versus vacuum freeze-drying on the protein quality of *Hippocampus abdominalis*—a commercially important cultured seahorse species—remain poorly understood.

Accordingly, this study aimed to investigate the effects of sun drying and vacuum freeze-drying on the nutritional composition and physicochemical properties of proteins in *Hippocampus abdominalis*, as well as to further elucidate the underlying molecular basis. Protein quality was evaluated by determining protein content, amino acid composition, solubility, surface hydrophobicity, water-holding capacity (WHC), and oil-holding capacity (OHC). In parallel, proteomic analysis was performed to identify differentially abundant proteins and to explore the potential associations between changes in protein composition and macroscopic quality attributes. This study provides a theoretical basis for a deeper understanding of the effects of drying methods on the protein quality of *Hippocampus abdominalis* and for its rational processing and utilization. However, this study only investigated sun drying and vacuum freeze-drying, and the effects of other commonly used drying methods remain to be explored.

## 2. Materials and Methods

### 2.1. Materials and Instruments

Twelve-month-old *Hippocampus abdominalis* were purchased from Rizhao Jiuyuan Aquaculture Company (Rizhao, China). Each whole seahorse weighed approximately 15 g and had a body length of approximately 15 cm. Amino acid standards, including aspartic acid (Asp), threonine (Thr), serine (Ser), glutamic acid (Glu), glycine (Gly), alanine (Ala), valine (Val), methionine (Met), isoleucine (Ile), leucine (Leu), tyrosine (Tyr), phenylalanine (Phe), lysine (Lys), histidine (His), arginine (Arg), and proline (Pro), were purchased from Shanghai Yuanye Bio-Technology Co., Ltd. (Shanghai, China) and used for amino acid analysis. Sulfuric acid, hydrochloric acid, and urea were purchased from Shanghai Aladdin Biochemical Technology Co., Ltd. (Shanghai, China) Coomassie Brilliant Blue R-250 was obtained from Sangon Biotech (Shanghai) Co., Ltd. (Shanghai, China), and bromophenol blue was purchased from Beijing Solarbio Science & Technology Co., Ltd. (Beijing China) Potassium sulfate/copper sulfate 15:1 catalytic tablets were purchased from Beijing Jinyuanxingke Technology Co., Ltd. (Beijing China) Acetonitrile and formic acid were both of mass spectrometry grade and were purchased from Thermo Fisher Scientific (Waltham, MA, USA).

The following major instruments were used in this study: an automatic Kjeldahl analyzer (Kjeltec 8400, FOSS, Hillerød, Denmark), an automatic amino acid analyzer (L-8900, Hitachi, Tokyo, Japan), microplate reader (SpectraMax 190, Molecular Devices, San Jose, CA, USA), vortex mixer (MS 3 digital, IKA, Staufen, Germany), and refrigerated centrifuge (5424R, Eppendorf, Hamburg, Germany).

### 2.2. Sample Preparation

Before the experiment, *Hippocampus abdominalis* samples were cleaned to remove surface impurities and blotted dry to remove surface moisture. The samples were then randomly divided into a sun-dried group and a freeze-dried group. Ten individuals were included in each group. Samples in the sun-dried group were naturally sun-dried in a well-ventilated, dust-protected environment and periodically turned until a constant weight was reached. The temperature, relative humidity, and light intensity were not artificially controlled during the sun-drying process, allowing the samples to remain under natural environmental conditions. The samples in the freeze-dried group were pre-frozen at −40 °C for 4 h and subsequently dried in a vacuum freeze dryer at −60 °C and 10 Pa for approximately 24 h until a constant weight was achieved. Constant weight was confirmed by periodic weighing until the sample weight remained stable. After grinding, approximately 30 g of sample was obtained from each group and stored in sealed bags under cool and dry conditions for subsequent analysis.

Briefly, 2.0 g of tissue powder from each freeze-dried and sun-dried *Hippocampus abdominalis* sample was accurately weighed and mixed with 5 mL of lysis buffer, followed by ultrasonic treatment. After sonication, the samples were centrifuged, and the resulting supernatants were collected. Subsequently, five volumes of acetone were added to each supernatant to precipitate the proteins for 2 h. The samples were then centrifuged again, and the supernatants were discarded. The resulting protein precipitates were collected and freeze-dried to obtain protein powders for subsequent physicochemical analyses.

### 2.3. Protein and Amino Acid Analysis

#### 2.3.1. Protein Content

The protein content of the samples was determined using the Kjeldahl method. An accurately weighed 0.2 g measure of each sample was digested with concentrated sulfuric acid in the presence of catalyst tablets containing potassium sulfate and copper sulfate at a ratio of 15:1, thereby converting organic nitrogen into ammonium nitrogen. The total nitrogen content was subsequently determined by distillation and titration. Protein content was calculated from the nitrogen content using a conversion factor of 6.25. Each sample was analyzed in triplicate, and the results were expressed as mean ± standard deviation.

The protein content was calculated as follows:

Protein content (%) = Total nitrogen content (%) × 6.25

#### 2.3.2. Hydrolyzed Amino Acids

The contents of hydrolyzed amino acids in the samples were determined using an automatic amino acid analyzer according to GB 5009.124-2016 (National Food Safety Standard—Determination of Amino Acids in Foods) [10]. The two dried samples were separately transferred into different hydrolysis tubes, and 5 mL of 6 mol/L hydrochloric acid was added before the tubes were sealed. The samples were then hydrolyzed in an oven at 110 °C for 24 h. After hydrolysis, the 5 mL hydrolysate was evaporated to dryness under a stream of nitrogen. The dried residue was reconstituted in 1 mL of sodium citrate buffer (pH 2.2) and subsequently analyzed using an automatic amino acid analyzer.

### 2.4. Physicochemical Properties

#### 2.4.1. Solubility

With slight modifications to the method described by Hu et al. [11], 20 mg of the protein sample was dispersed in 1 mL of distilled water and centrifuged at 10,000× *g* for 30 min at 4 °C. The protein concentration in the supernatant was determined using the Bradford assay, with bovine serum albumin (BSA) as the standard. Protein solubility was calculated as the percentage of protein in the supernatant relative to the total protein content of the sample. All measurements were performed in triplicate, and the results were expressed as the mean of three determinations.

#### 2.4.2. Isoelectric Point

Phosphate buffer solutions with pH values of 2.5, 3.5, 4.5, 5.5, 6.5, and 7.5 were accurately prepared. We accurately weighed 20 mg of the protein sample into a 10 mL centrifuge tube, followed by the addition of 5 mL of buffer solution at the corresponding pH. The mixtures were thoroughly homogenized and centrifuged at 8000 rpm for 20 min to separate the supernatant from the precipitate. The protein concentration in the supernatant was determined using the Coomassie Brilliant Blue method. A standard curve was established using BSA solutions of different concentrations. After the addition of the Coomassie Brilliant Blue reagent, the absorbance was measured at 595 nm, and the protein concentration was calculated from the standard curve. The pH of the buffer solution corresponding to the lowest protein concentration was considered the isoelectric point of *Hippocampus abdominalis* proteins.

#### 2.4.3. Surface Hydrophobicity

With slight modifications to the method described by Chu et al. [12], we adjusted the concentration of the protein solution to 5 mg/mL. We took 1 mL of the protein solution and mix it with 200 μL of bromophenol blue with a concentration of 1 mg/mL. We then vortexed the mixture for 3 min and then centrifuge it at 8000× *g* in a low-temperature centrifuge. The supernatant obtained after centrifugation was diluted 10-fold with PBS solution containing 0.6 mol/L KCl, 0.02 mol/L KH_2_PO_4_, and 0.02 mol/L K_2_HPO_4_ (pH 7.0), and the absorbance was measured at 595 nm. A mixture of 1 mL of PBS solution and 200 μL of bromophenol blue solution was used as the control, with all other procedures performed identically. Each sample was analyzed in triplicate, and the surface hydrophobicity of myosin was calculated according to the equation bromophenol blue bound (μg) = (A0 − A)/A × 200, where A0 and A represent the absorbance values of the blank control and the sample mixture, respectively.

#### 2.4.4. WHC and OHC

With slight modifications to the method described by Jin et al. [13], 10 mg of protein powder was placed in a centrifuge tube, followed by the addition of 1 mL of sunflower oil or distilled water. The mixture was vortexed for 30 s and allowed to stand at room temperature for 30 min. Subsequently, the mixture was centrifuged at 8000 rpm for 20 min, and the resulting precipitate was collected. The OHC and WHC were calculated according to the equation OHC/WHC (g/g) = (m2 − m1)/m0, where m2 represents the total mass of the precipitate and centrifuge tube after separation of the oil or water (g), m1 represents the mass of the empty centrifuge tube (g), and m0 represents the mass of the protein powder (g).

### 2.5. Proteomic Analysis

The samples were first subjected to solubilization and protein extraction. An equal amount of each sample was transferred into a 2 mL grinding tube and thoroughly homogenized. The homogenates were then incubated at 95 °C for 5 min with shaking at 1500 rpm. Subsequently, the samples were centrifuged at 14,000× *g* for 10 min at 4 °C, and the supernatants were collected. Protein concentrations were quantified using a BCA Protein Assay Kit (Bio-Rad, Hercules, CA, USA).

### 2.6. Liquid Chromatography–Tandem Mass Spectrometry (LC–MS/MS) Analysis

Data were acquired using Thermo Xcalibur 4.7 software (Thermo Fisher Scientific, Waltham, MA, USA). Chromatographic separation was performed on a Vanquish Neo system (Thermo Fisher Scientific, USA) equipped with a homemade column (15 cm × 100 μm, 1.7 μm). Mass spectrometric analysis was conducted using an Astral mass spectrometer (Thermo Fisher Scientific, USA). The chromatographic separation time was 8 min. Mobile phase A consisted of 2% acetonitrile and 0.1% formic acid, while mobile phase B consisted of 80% acetonitrile and 0.1% formic acid. The gradient elution program was as follows: 0–1 min, 92% A/8% B; 1–5.5 min, the proportion was changed to 83% A/17% B; 5.5–7 min, to 45% A/55% B; and 7–8 min, to 1% A/99% B. Samples separated by nano-high-performance liquid chromatography (nano-HPLC) were analyzed using an ASTRAL mass spectrometer (Thermo Fisher Scientific, Bremen, Germany) in data-independent acquisition (DIA) mode. Mass spectrometric analysis was performed in positive-ion mode with an ion source voltage of 2.2 kV. Both MS1 and MS/MS spectra were acquired using the mass spectrometer. The MS1 scan range was 380–980 *m*/*z*, and the MS/MS scan range was 150–2000 *m*/*z*.

The database used for the proteomic analysis was uniprot-taxonomy-72046-unique. Oxidation (M) and carbamidomethylation (C) were selected as modifications for the database search. Label-free quantification (LFQ) was applied with a minimum ratio count of 1. The “Match between runs” option was enabled, with a match time window of 0.7 min. Both the false discovery rate (FDR) and q-value thresholds were set at <0.05.

### 2.7. Statistical Analysis

All data were presented as the mean ± standard error of the mean (SEM) based on at least three independent replicates. Each experiment was performed with three biological replicates (*n* = 3). Statistical analyses were performed using IBM SPSS Statistics software (Version 27). Differences among groups were analyzed by one-way analysis of variance (ANOVA), followed by the least significant difference (LSD) test for multiple comparisons.

## 3. Results and Discussion

### 3.1. Protein and Amino Acid Analysis of Hippocampus abdominalis Under Different Drying Methods

Protein is one of the major nutritional components of *Hippocampus abdominalis* and serves as an important structural basis for muscle and connective tissues, as well as for various functional proteins. As shown in Table 1, the crude protein content of sun-dried *Hippocampus abdominalis* was 68.57 ± 0.53%, which was higher than that of the freeze-dried samples (61.86 ± 1.84%). This difference may be related to the relative concentration of protein resulting from the loss of moisture and other components during sun drying [14]. The moisture content measured after drying the fresh seahorses was 72.52 ± 0.31%. Nevertheless, differences in protein content should not be interpreted solely in terms of concentration effects, as drying conditions may also induce changes in protein structure and chemical state. In particular, prolonged exposure to heat and oxygen during sun drying may promote protein denaturation and oxidation, while endogenous proteolytic activity may contribute to protein degradation. These processes could influence the overall protein quality and physicochemical properties of the samples, although their specific contributions were not directly determined in the present study. Further studies involving protein oxidation, structural characterization, and proteolysis-related analyses are therefore warranted.

Amino acid composition is an important indicator of the nutritional quality of seahorses and is closely associated with the processing method. The amino acid contents of *Hippocampus abdominalis* subjected to different drying treatments are shown in Table 1. The total amino acid content of the freeze-dried samples (58.79 ± 0.08%) was significantly higher than that of the sun-dried samples (48.87 ± 0.04%), suggesting that vacuum freeze-drying was more favorable for maintaining amino acid levels [15,16].

From a nutritional perspective, amino acids can be classified into essential amino acids (EAAs) and non-essential amino acids (NEAAs). The total EAA contents of the freeze-dried and sun-dried *Hippocampus abdominalis* samples were 20.29% and 19.12%, respectively, while the corresponding total NEAA contents were 38.48% and 29.77%, respectively. This indicates that the decrease in total amino acid content was mainly attributable to the reduction in NEAAs, among which Gly and Pro showed particularly pronounced decreases. Notably, Gly and Pro are characteristic amino acids of collagen [17]: Gly is an important precursor for protein synthesis and participates in protein synthesis and energy metabolism, whereas Pro plays a key role in collagen synthesis, wound healing, and immune responses [18]. Changes in their contents may be associated with structural alterations of collagen and connective tissue proteins in seahorses during sun drying. In addition, the slow migration of moisture toward the sample surface during sun drying may be accompanied by the migration of some water-soluble nitrogen-containing components [19]. Meanwhile, tissue shrinkage and densification caused by the evaporation of liquid water may reduce reagent penetration and amino acid release efficiency during acid hydrolysis. Therefore, the marked decrease in total amino acid content suggests that the adverse effects of thermal processing on most amino acids cannot be ignored.

Compared with prolonged drying under ambient conditions, the low-temperature and vacuum conditions employed during freeze-drying reduce prolonged thermal and oxidative exposure, which may contribute to better preservation of amino acid-containing protein components. Therefore, the contrasting trends in crude protein and total amino acid contents observed in the present study further demonstrate that crude protein content alone is insufficient to comprehensively evaluate protein quality after drying.

### 3.2. Physicochemical Properties of Proteins in Hippocampus abdominalis Under Different Drying Methods

Protein is a major nutritional component of *Hippocampus abdominalis*. Changes in protein molecular structure, conformational stability, and intermolecular interactions during drying may further affect its physicochemical properties and, consequently, its processing and utilization characteristics. Therefore, comparing the effects of different drying methods on the physicochemical properties of *Hippocampus abdominalis* proteins may help elucidate changes in protein structure during processing and evaluate their potential utilization value.

#### 3.2.1. Solubility

**Figure 1:**
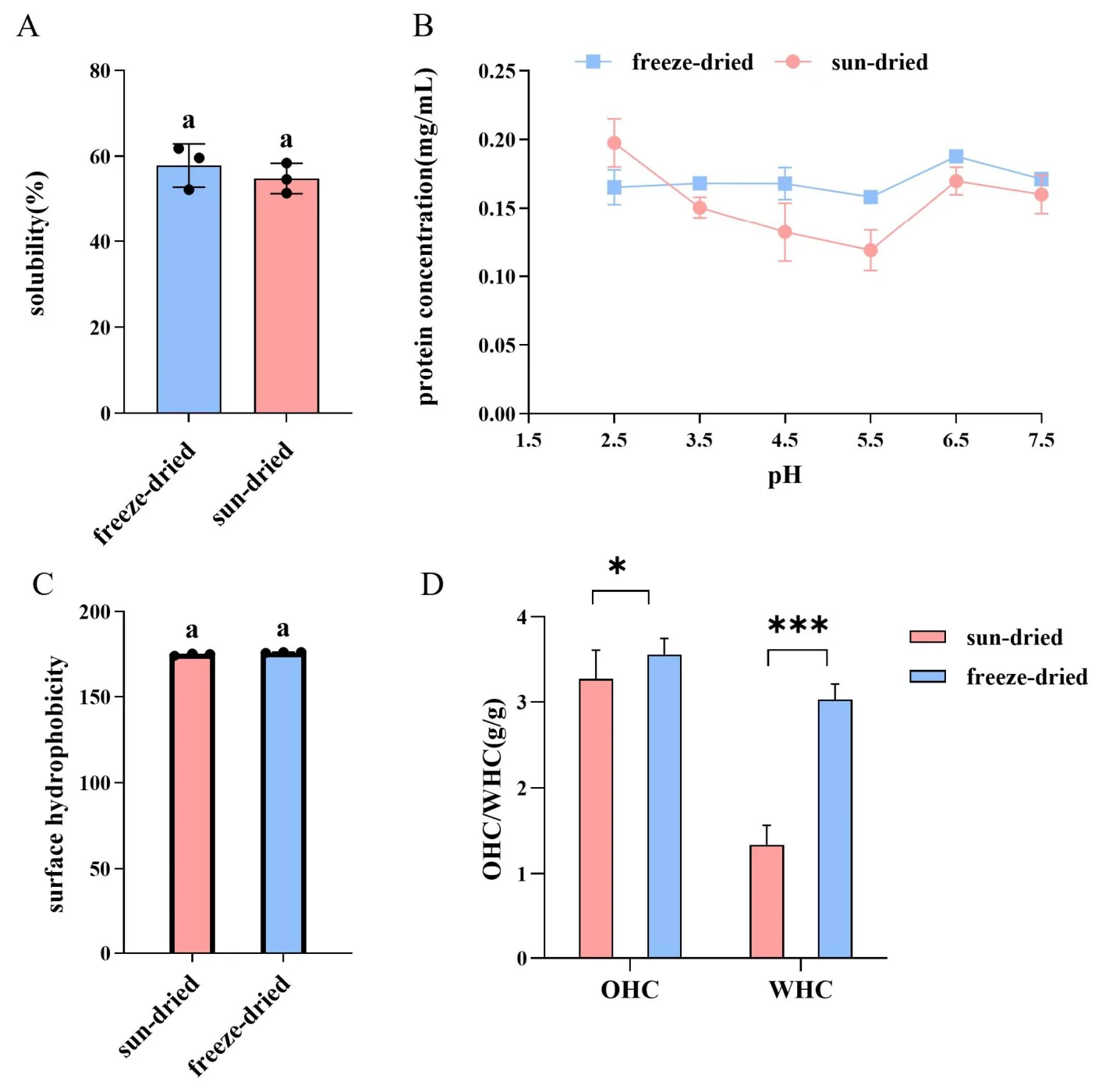
Physicochemical properties of proteins in *Hippocampus abdominalis* subjected to different drying methods: (**A**) protein solubility; (**B**) protein isoelectric point; (**C**) protein surface hydrophobicity; (**D**) WHC and OHC. Bars sharing the same lowercase letter (a) are not significantly different (*p* > 0.05). Asterisks indicate significant differences between the indicated groups: * *p* < 0.05 and *** *p* < 0.001.

Protein solubility is an important indicator of its dispersibility and hydration capacity in aqueous systems and can, to some extent, indirectly reflect the degree of protein denaturation, oxidation, and aggregation during drying. As shown in Figure 1A, the protein solubilities of the freeze-dried and sun-dried *Hippocampus abdominalis* samples were approximately 58% and 55%, respectively, with no significant difference between the two groups. Compared with sun drying, freeze-drying reduces prolonged protein exposure to relatively high temperatures and oxygen levels and produces a relatively loose and porous structure, thereby facilitating protein rehydration and resolubilization [20]. However, ice crystal formation, interfacial interactions, and dehydration stress during freeze-drying may still induce partial protein denaturation and oxidation [21]. This may explain why the protein solubility of the freeze-dried group was only slightly higher than that of the sun-dried group, with no significant difference observed between the two groups.

#### 3.2.2. Isoelectric Point Analysis

The isoelectric point is the pH at which a protein molecule carries no net electrical charge and is an important parameter affecting protein solubility and aggregation behavior. When the pH of the system approaches the isoelectric point, electrostatic repulsion between protein molecules decreases, while hydrophobic interactions become more prominent, thereby promoting protein aggregation and precipitation and resulting in minimum protein solubility [22]. As shown in Figure 1B, the solubility of proteins from both freeze-dried and sun-dried *Hippocampus abdominalis* initially decreased and then increased with increasing pH, reaching a minimum at approximately pH 5.5. This indicates that the apparent isoelectric point of the proteins in both groups was approximately pH 5.5. The absence of an obvious shift in the isoelectric point suggests that freeze-drying and sun drying had relatively limited effects on the overall charge-balance characteristics of *Hippocampus abdominalis* proteins. However, differences in protein solubility between the two groups at different pH values indicate that the drying method may still affect the degree of protein aggregation and intermolecular interactions.

#### 3.2.3. Surface Hydrophobicity

The hydrophobicity of the protein surface is an important indicator for assessing the degree of protein oxidation. As shown in Figure 1C, the surface hydrophobicity values of proteins from sun-dried and freeze-dried *Hippocampus abdominalis* were 174.879 ± 0.659 and 176.111 ± 0.260, respectively. The slightly higher value observed in the freeze-dried group may be attributed to freezing and dehydration during freeze-drying, which may induce subtle conformational changes and expose some hydrophobic groups that were originally buried within the protein structure. In contrast, protein aggregation may occur during sun drying, causing some hydrophobic regions to become re-embedded within the aggregates. Despite these trends, the difference between the two groups was relatively small, suggesting that the overall effects of the two drying methods on protein surface hydrophobicity were limited.

#### 3.2.4. WHC and OHC

WHC and OHC are important indicators of the processing functionality of proteins, reflecting the ability of protein powders to retain water and oil, respectively, under the action of external forces. As shown in Figure 1D, both the OHC and WHC were higher in the freeze-dried group than in the sun-dried group. The higher WHC of the freeze-dried samples may be associated with the loose and porous structure formed during freeze-drying. During freeze-drying, water is removed by sublimation from the frozen state, which may partly alleviate structural shrinkage caused by liquid-water migration and capillary pressure, thereby preserving more pores that facilitate water penetration and retention in the dried samples [23]. Protein OHC is determined not only by hydrophobic interactions between nonpolar amino acid side chains and oil molecules but also by particle size, pore structure, bulk density, and the aggregation state of proteins [24]. During freeze-drying, sublimation of ice crystals can preserve pores and channels within the sample, resulting in a relatively loose powder structure that facilitates oil penetration into the particles and promotes its physical retention through capillary forces and the particle network [25]. In contrast, the continuous migration of moisture and tissue shrinkage during sun drying may lead to a more compact particle structure, thereby reducing the internal space available for oil penetration and retention.

Interestingly, the effects of drying methods on the different physicochemical properties were not entirely consistent. Although the freeze-dried samples exhibited higher WHC and OHC, no significant differences were observed in protein solubility or surface hydrophobicity between the two groups. This suggests that the influence of drying on *Hippocampus abdominalis* protein functionality was property-dependent rather than uniform. Protein solubility is affected by multiple factors, including protein–protein interactions, aggregation state, surface charge, and molecular conformation, whereas surface hydrophobicity mainly reflects the accessibility of hydrophobic regions. In contrast, WHC and OHC are additionally influenced by the physical characteristics of the dried matrix, including porosity, particle structure, and capillary retention [26]. Therefore, the higher WHC and OHC of the freeze-dried samples do not necessarily require substantial changes in overall protein solubility or surface hydrophobicity. The porous structure generated by ice sublimation during freeze-drying may have contributed to enhanced physical retention of water and oil.

### 3.3. Proteomic Analysis

#### 3.3.1. Quality Assessment of Mass Spectrometry Data

**Figure 2:**
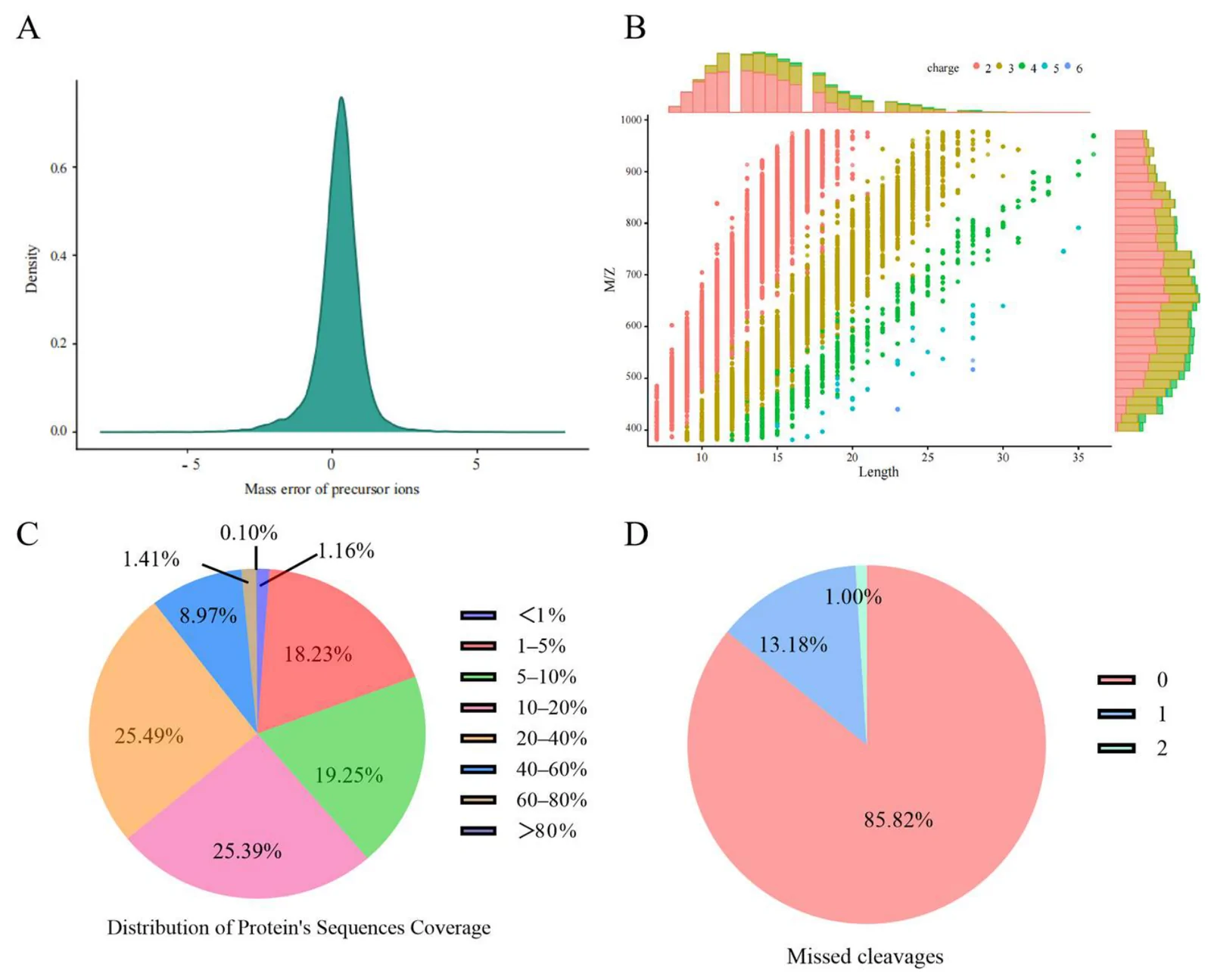
Quality assessment of mass spectrometry data: (**A**) mass error distribution of detected precursor ions; (**B**) length distribution of identified peptides; (**C**) sequence coverage distribution of identified proteins; (**D**) distribution of missed-cleavage peptides.

Based on the above-mentioned differences in protein content, amino acid composition, and physicochemical properties, this study employed proteomic approaches to conduct an in-depth analysis of the two groups of samples, with the aim of further elucidating the molecular basis underlying the effects of different drying methods on *Hippocampus abdominalis* proteins. First, the reliability of the mass spectrometry identification data was evaluated by performing quality-control analyses of precursor ion mass errors, peptide length and charge distributions, missed cleavage events, and protein sequence coverage. As shown in Figure 2A, the precursor ion mass errors were predominantly centered around 0 ppm and exhibited a relatively narrow distribution, indicating the good mass accuracy of the mass spectrometric measurements. As shown in Figure 2B, the identified peptides were mainly distributed within a length range of approximately 7–30 amino acids, with most peptides containing 8–20 amino acids. The predominant peptide charge states were 2+ and 3+, and the mass-to-charge ratios (*m*/*z*) were mainly concentrated between 400 and 1000, which is consistent with the general characteristics of tryptic peptides and high-resolution mass spectrometric detection. As shown in Figure 2C, the missed-cleavage analysis revealed that peptides with no missed cleavage accounted for 85.82%, those with one missed cleavage accounted for 13.18%, and those with two missed cleavages accounted for only 1.00%. Thus, peptides with zero or one missed cleavage together accounted for 99.00%, indicating that enzymatic digestion of the samples was relatively complete. As shown in Figure 2D, analysis of protein sequence coverage revealed that proteins with sequence coverage of 10–20% and 20–40% accounted for 25.39% and 25.49%, respectively, representing the predominant coverage ranges. Proteins with sequence coverage above 10% collectively accounted for 61.36%, while 80.61% of the proteins exhibited coverage above 5%. In contrast, proteins with sequence coverage below 1% accounted for only 1.16%. Overall, the precursor ion mass errors, peptide lengths, charge-state distributions, and missed-cleavage profiles were all within reasonable ranges, and the overall protein sequence coverage was satisfactory, indicating that the mass spectrometry identification data obtained in this study were reliable.

#### 3.3.2. Differentially Abundant Protein Analysis

**Figure 3:**
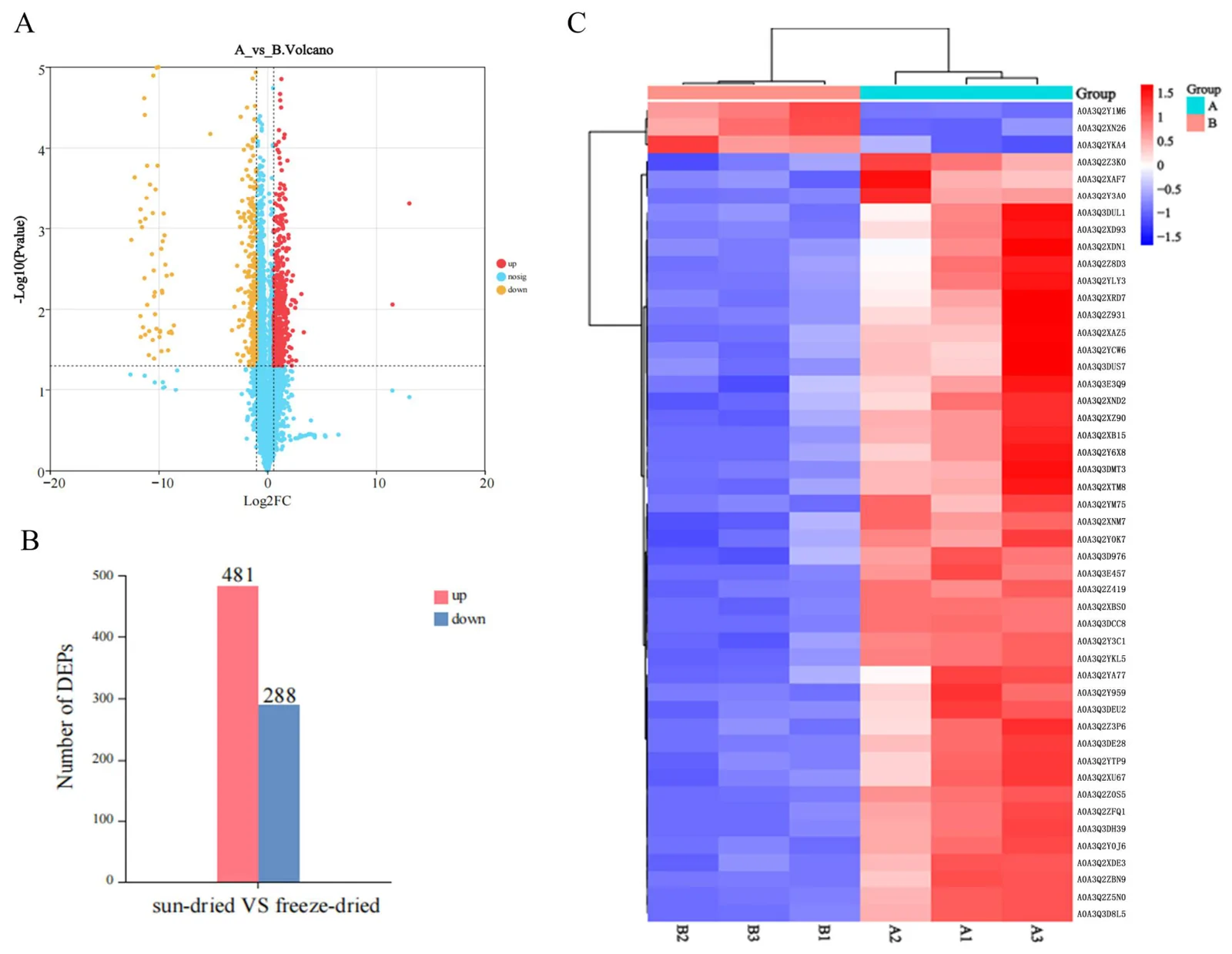
Screening and clustering analysis of differentially abundant proteins in sun-dried and freeze-dried *Hippocampus abdominalis*: (**A**) volcano plot of differentially abundant proteins; (**B**) number of differentially abundant proteins; (**C**) hierarchical clustering heatmap of representative differentially abundant proteins.

LC–MS/MS analysis revealed that different drying methods markedly affected the relative abundance distribution of the *Hippocampus abdominalis* proteome. As shown in the volcano plot (Figure 3A), most proteins were clustered around a Log_2_FC value of 0, whereas a considerable number of proteins exceeded the differential screening thresholds, indicating marked changes in the relative abundance of a subset of proteins between the sun-dried and freeze-dried samples. Further statistical analysis (Figure 3B) identified a total of 769 differentially abundant proteins between the two groups, of which 481 showed higher relative abundance in the sun-dried group and 288 showed higher relative abundance in the freeze-dried group. The greater number of proteins with higher relative abundance in the sun-dried group showed a certain degree of consistency with its higher crude protein content. These results suggest that different drying methods may selectively affect the stability and retention of different proteins, thereby resulting in differences in protein composition and relative abundance. Hierarchical clustering analysis (Figure 3C) further showed that the sun-dried samples (A1–A3) and freeze-dried samples (B1–B3) clustered into distinct branches. Samples within each group exhibited generally consistent abundance patterns, whereas a clear separation was observed between the two groups, indicating that the drying method affected the protein composition and abundance of *Hippocampus abdominalis*. This may be related to differences between the two drying methods in terms of temperature, dehydration rate, oxygen exposure, and protein structural stability. Therefore, the drying method is an important processing factor affecting the protein composition and relative abundance distribution of *Hippocampus abdominalis*.

#### 3.3.3. Identification of Proteins Affected by Different Drying Methods in *Hippocampus abdominalis*

To explore the effects of the drying method more comprehensively, by screening the proteins with a log2 fold change greater than 2.0 or less than −1.0, and a *p*-value less than 0.05. A total of 307 proteins showed significant changes, as shown in Table S1. Among these, 19 proteins exhibited higher relative abundance in the sun-dried *Hippocampus abdominalis* samples, whereas 288 proteins exhibited higher relative abundance in the freeze-dried samples. These results indicate that a substantially larger number of proteins were present at higher relative abundance in the freeze-dried samples than in the sun-dried samples.

Among the proteins differing between the freeze-dried and sun-dried *Hippocampus abdominalis* samples, those with higher relative abundance in the sun-dried samples mainly included proteins associated with cell adhesion and tissue structure, redox processes, metabolism, and protein homeostasis. Among these, Tenomodulin (A0A3Q3DJN1), Integrin subunit beta like 1 (A0A3Q3D3Y8), Contactin 2 (A0A3Q2XSV1), and Filamin A interacting protein 1 (A0A3Q2YDM0) are mainly involved in the maintenance of tissue structure and cell–matrix interactions; Tenomodulin (A0A3Q3DJN1) is considered an important protein associated with connective tissues such as tendons and ligaments [27]. Redox-related proteins such as catalase (A0A3Q2YLP6) are involved in reactive oxygen species scavenging and redox homeostasis, whereas 3-hydroxyisobutyrate dehydrogenase (A0A3Q2Z2T1) participates in valine catabolism [28]. The higher relative abundance in the sun-dried group may be related to differences in protein stability during the drying process and selective retention. Studies on sun-dried aquatic products have demonstrated that protein oxidation and degradation increase during drying, accompanied by enhanced hydrophobic interactions and disulfide bonds [29].

In addition, proteins with higher relative abundance in the freeze-dried *Hippocampus abdominalis* samples included myofibrillar proteins, proteins associated with amino acid metabolism and transport, and mitochondrial energy metabolism proteins. The most pronounced differences included myosin tail domain-containing protein (A0A3Q2YG83), cytochrome c oxidase subunit NDUFA4 (A0A3Q3E680), catalase (A0A4D6C7V6), microsomal glutathione S-transferase 1 (A0A3Q2Y374), and alanine–glyoxylate aminotransferase (A0A3Q2ZD74). This may be related to the rapid reduction in water activity under the low-temperature and vacuum conditions of freeze-drying, thereby reducing protein oxidation, denaturation, aggregation, and degradation. Studies on *Antarctic krill* have shown that the abundances of skeletal muscle actin, titin, paramyosin, and several metabolic enzymes were higher in the freeze-dried group than in the hot-air-dried group, suggesting that freeze-drying is more favorable for maintaining a relatively intact tissue proteome [30]. Therefore, freeze-drying provided better preservation of muscle structural proteins and certain proteins related to antioxidant activity and amino acid metabolism in *Hippocampus abdominalis*, whereas the continuous oxidation and structural changes occurring during sun drying may be important factors contributing to their lower relative abundance.

The above proteomic results were consistent with the aforementioned trends in amino acid composition and protein physicochemical properties. The freeze-dried group exhibited a higher total amino acid content, particularly higher levels of Gly, Pro, and Ala, together with greater WHC and OHC. Combined with the higher relative abundance of multiple myofibrillar proteins and some metabolism-related proteins in the freeze-dried group, it can be inferred that low-temperature vacuum conditions may reduce protein oxidation, aggregation, and degradation during drying, thereby maintaining protein structural integrity and the effective exposure of hydrophilic groups and enhancing interactions between proteins and water molecules. Thus, the proteomic results provide further molecular-level evidence supporting the higher amino acid retention levels and greater WHC and OHC of the freeze-dried samples. Notably, no significant differences were observed in protein solubility or surface hydrophobicity between the two groups, indicating that although different drying methods significantly altered the relative abundance and composition of specific proteins, their effects on the overall hydration state of the protein system and the degree of hydrophobic-group exposure were relatively limited. This further suggests that the drying process exerts a certain degree of selective effect on different proteins.

## 4. Conclusions

This study demonstrates that sun drying and vacuum freeze-drying differentially modulate the protein quality of H. abdominalis through distinct mechanisms. Although the sun-dried group had a higher crude protein content, vacuum freeze-drying was more favorable for the retention of amino acids, particularly Gly and Pro, and resulted in greater WHC and OHC. However, the two drying methods had no significant effects on protein solubility or surface hydrophobicity, indicating that their effects on the overall molecular exposure state of the protein system were relatively limited. Proteomic analysis further revealed that most muscle structural proteins and metabolism-related proteins exhibited higher relative abundance in the freeze-dried group, whereas some redox-related and cell-adhesion proteins were relatively enriched in the sun-dried group. These results indicate that freeze-drying may help maintain the integrity of protein composition, whereas sun drying may alter protein distribution through oxidation and aggregation. In summary, freeze-drying was superior to sun drying in maintaining the nutritional quality and functional properties of *Hippocampus abdominalis* proteins. However, given its relatively high energy consumption and associated costs, the choice of drying method in practical applications should be made comprehensively based on product positioning and processing scale.

## Figures and Tables

**Table 1:** Protein and amino acid contents of *Hippocampus abdominalis* subjected to different drying methods.

Category	Parameter	Freeze-Dried (%)	Sun-Dried (%)
Basic composition	Protein content	61.86 ± 1.84 ^b^	68.57 ± 0.53 ^a^
EAAs	Thr	2.27 ± 0.01 ^a^	2.12 ± 0.01 ^b^
	Val	2.87 ± 0.01 ^a^	2.62 ± 0.01 ^b^
	Met	1.56 ± 0.02 ^a^	1.36 ± 0.02 ^b^
	Ile	2.07 ± 0.01 ^a^	2.08 ± 0.02 ^a^
	Leu	3.58 ± 0.01 ^b^	3.64 ± 0.01 ^a^
	Phe	2.15 ± 0.01 ^a^	1.94 ± 0.01 ^b^
	Lys	4.44 ± 0.01 ^a^	4.11 ± 0.03 ^b^
	His	1.35 ± 0.00 ^a^	1.25 ± 0.01 ^b^
	ΣEAAs	20.29	19.12
NEAAs	Ser	2.62 ± 0.01 ^a^	2.29 ± 0.01 ^b^
	Glu	6.98 ± 0.02 ^a^	6.16 ± 0.02 ^b^
	Gly	8.66 ± 0.02 ^a^	5.15 ± 0.02 ^b^
	Ala	4.64 ± 0.00 ^a^	3.50 ± 0.02 ^b^
	Asp	4.99 ± 0.01 ^b^	4.46 ± 0.01 ^a^
	Tyr	1.55 ± 0.01 ^b^	1.62 ± 0.01 ^a^
	Arg	4.66 ± 0.01 ^a^	3.65 ± 0.02 ^b^
	Pro	4.38 ± 0.01 ^a^	2.94 ± 0.01 ^b^
	ΣNEAAs	38.48	29.77
	Total AA	58.79 ± 0.08 ^a^	48.87 ± 0.04 ^b^

Results were expressed as Mean ± standard deviations; *n* = 3 for all treatments. Values with different superscript letters (a, b) within the same row indicate significant differences between drying methods (*p* < 0.05). Note: EAAs refer to essential amino acids, while NEAAs refer to non-essential amino acids.

## Data Availability

The original contributions presented in this study are included in the article/Supplementary Material. Further inquiries can be directed to the corresponding authors.

## Supplementary Materials

The following supporting information can be downloaded at https://www.mdpi.com/article/10.3390/foods15183236/s1. Table S1. Differentially abundant proteins with increased abundance in sun-dried *Hippocampus abdominalis* compared with freeze-dried samples; Table S2. Differentially abundant proteins with decreased abundance in sun-dried *Hippocampus abdominalis* compared with freeze-dried samples.

## Abbreviations

The following abbreviations are used in this manuscript:

Asp	Aspartic acid
Thr	Threonine
Ser	Serine
Glu	Glutamic acid
Gly	Glycine
Ala	Alanine
Val	Valine
Met	Methionine
Ile	Isoleucine
Leu	Leucine
Tyr	Tyrosine
Phe	Phenylalanine
Lys	Lysine
His	Histidine
Arg	Arginine
Pro	Proline
WHC	Water-holding capacity
OHC	Oil-holding capacity
LC–MS/MS	Liquid chromatography–tandem mass spectrometry
EAAs	Essential amino acids
NEAAs	Non-essential amino acids
BSA	Bovine serum albumin
nano-HPLC	nano-high-performance liquid chromatography
DIA	Data-independent acquisition
