# Supplementary material for: Effects of Sun Drying Versus Vacuum Freeze-Drying on the Protein Quality of Hippocampus abdominalis: Nutritional Composition, Physicochemical Properties, and Proteomic Profiles
Source: Foods. 2026 Sep 13;15(18):3236. doi: 10.3390/foods15183236 (PMC13606909; doi:10.3390/foods15183236)
Supplement: Supplementary file 1 [file foods-15-03236-s001.zip › foods-4532876-supplementary.pdf]

Table S1. Differentially abundant proteins with increased abundance in sun-dried *Hippocampus abdominalis* compared with freeze-dried samples

UniProt accession	Protein Name	Log2FC (sun-dried/freeze-dried)	Abundance change	P-value
A0A3Q3E5K7	Apolipoprotein M	2.149	up	8.83E-03
A0A3Q2YJB8	E3 ubiquitin-protein ligase CBL	2.6172	up	8.21E-03
A0A3Q3DJN1	Tenomodulin	2.0393	up	3.85E-02
A0A3Q2YRD9	Protein S100-A16-like	3.129	up	6.48E-03
A0A3Q2Z2T1	3-hydroxyisobutyrate dehydrogenase	3.3506	up	1.93E-02
A0A3Q3DH73	5,6-dihydroxyindole-2-carboxylic acid oxidase	2.2075	up	3.03E-02
A0A3Q2XYY6	OX-2 membrane glycoprotein-like	2.561	up	9.60E-03
A0A3Q2Y4W9	Monocarboxylate transporter 1	2.2822	up	7.67E-03
A0A3Q2YLP6	Catalase	13.0587	up	4.84E-04
A0A3Q3D6U1	Melanoregulin-like	2.0137	up	2.93E-02
A0A3Q2XAK0	Protein NipSnap homolog 1-like	2.2078	up	4.49E-03
A0A3Q2ZFI5	Ras-related protein Rab	2.3491	up	3.81E-03
A0A3Q2XYC9	BPTI/Kunitz inhibitor domain-containing protein	2.2446	up	4.97E-02
A0A3Q2XJH5	5,6-dihydroxyindole-2-carboxylic acid oxidase	2.6483	up	4.30E-02
A0A3Q3DKQ7	Dehydrogenase/reductase SDR family member on chromosome X-like	2.2477	up	4.26E-02
A0A3Q3D3Y8	Integrin subunit beta like 1	2.4794	up	7.76E-03
A0A3Q2XSV1	Contactin 2	2.3359	up	1.84E-02
A0A3Q2YDM0	Filamin A interacting protein 1	11.5121	up	8.73E-03
A0A3Q2ZI12	peptidylprolyl isomerase	2.0662	up	2.09E-02

Table S2. Differentially abundant proteins with decreased abundance in sun-dried *Hippocampus abdominalis* compared with freeze-dried samples

UniProt accession	Protein Name	Log2FC (sun-dried/freeze-dried)	Abundance change	P-value
A0A3Q2YKA4	Myosin regulatory light chain 2, skeletal muscle isoform-like	-1.058	down	3.14E-03
A0A3Q2XN26	Myosin tail domain-containing protein	-1.119	down	9.58E-04
A0A3Q2Y1M6	Myosin heavy chain, fast skeletal muscle	-1.5973	down	2.91E-04
A0A3Q2XGH2	ADP/ATP translocase	-1.0227	down	1.14E-02
A0A4D6C7V6	Catalase	-1.3548	down	3.893E-6

A0A3Q2Y746	Myosin-11	-1.1263	down	3.64E-03
A0A3Q2XG74	S-adenosylmethionine synthase	-1.4262	down	8.739E-5
A0A3Q2YT57	Troponin I, fast skeletal muscle-like	-1.3357	down	7.33E-03
A0A3Q2XGX9	Cysteine dioxygenase	-1.2406	down	5.60E-04
A0A3Q2ZJW4	Neuronal acetylcholine receptor subunit alpha-7-like	-1.2324	down	1.43E-02
A0A3Q2XU59	Calcium-binding mitochondrial carrier protein Aralar1-like	-1.0125	down	4.67E-03
A0A3Q2Y7M7	Gamma-crystallin M3-like	-2.7809	down	2.89E-03
A0A3Q2YA90	Alpha-crystallin A chain	-1.7269	down	3.32E-02
A0A3Q3DIU0	Gamma-crystallin M1-like	-2.5666	down	1.36E-02
A0A3Q2XHI0	Ig-like domain-containing protein	-1.1122	down	6.96E-03
A0A3Q2XRU5	Galectin	-1.9902	down	1.90E-03
A0A3Q2YEC6	Hexose-6-phosphate dehydrogenase/glucose 1-dehydrogenase	-1.3302	down	3.88E-03
A0A3Q2XPS2	Alpha-2-HS-glycoprotein-like	-1.4181	down	4.58E-04
A0A3Q2XFS1	Cadherin 17	-1.0957	down	4.66E-03
A0A3Q3E0B9	Multifunctional fusion protein	-1.0297	down	1.88E-04
A0A1Q1NLK4	Myosin heavy chain 6	-1.4309	down	6.95E-04
A0A3Q2Z1I9	Cytochrome b-c1 complex subunit 2, mitochondrial-like	-1.0345	down	1.14E-03
A0A141MJB8	Ferritin	-1.9219	down	1.76E-04
A0A141MJB7	Ferritin	-1.1854	down	4.98E-03
A0A3Q2XV45	Slow myosin heavy chain 2	-1.2039	down	4.08E-02
A0A3Q3DVW0	Mitochondrial 2-oxoglutarate/malate carrier protein	-1.0374	down	2.97E-02
A0A3Q2YDN4	Alpha-crystallin B chain-like	-1.7568	down	1.23E-02
A0A3Q2XKU8	Uncharacterized LOC109520244	-1.138	down	2.041E-5
A0A3Q2XQG6	Asialoglycoprotein receptor 1-like	-1.2626	down	4.50E-03
A0A3Q2Y8P6	Tropomyosin alpha-1 chain	-1.0113	down	4.16E-02
A0A3Q2XVT7	SH3 domain-binding glutamic acid-rich-like protein	-1.2122	down	3.35E-02
A0A3Q2Z515	Galectin	-1.3548	down	1.96E-02
A0A3Q2Z455	Myosin-2-like	-1.3868	down	7.43E-03
A0A3Q3E848	Microsomal triglyceride transfer protein	-1.3456	down	6.782E-5
A0A3Q3DS31	Arachidonate 12-lipoxygenase, 12R-type-like	-1.1976	down	3.43E-5
A0A3Q2XL90	Reticulon	-1.0469	down	1.76E-04
A0A3Q2Y4V7	Cytochrome P450 3A	-1.0221	down	6.308E-5
A0A3Q2YTP8	Cytochrome c oxidase subunit 5B, mitochondrial	-1.0422	down	2.08E-03
A0A3Q3E2M3	Myosin light chain kinase, smooth	-1.125	down	2.52E-04

	muscle-like			
A0A3Q2XGW9	Gamma-crystallin S	-1.3835	down	3.81E-02
A0A3Q3DUB8	Myosin N-terminal SH3-like domain-containing protein	-1.8916	down	3.98E-03
A0A3Q2XY01	Bactericidal permeability-increasing protein	-1.4647	down	7.88E-03
A0A3Q2YG83	Myosin tail domain-containing protein	-2.0339	down	6.20E-03
A0A3Q2Y591	Protein-cysteine N-palmitoyltransferase HHAT-like protein	-1.1707	down	5.94E-04
A0A3Q2ZD74	Alanine--glyoxylate aminotransferase	-1.8704	down	2.169E-5
A0A3Q2YGV6	Troponin I, slow skeletal muscle-like	-1.6538	down	6.92E-03
A0A3Q3DIE1	Gamma-crystallin M3-like	-2.4266	down	5.57E-03
A0A3Q2Y374	Microsomal glutathione S-transferase 1	-1.5113	down	4.59E-04
A0A3Q2YRB2	Trimeric intracellular cation channel type A	-1.0003	down	2.55E-03
A0A3Q2ZB94	Myozenin-1-like	-1.0505	down	3.01E-03
A0A3Q2YCC2	Sodium/potassium-transporting ATPase subunit beta	-1.737	down	6.01E-03
A0A3Q3D6B2	Transmembrane protein 14C	-1.3544	down	2.85E-03
A0A3Q3DM77	Ezrin	-1.1498	down	6.94E-04
A0A3Q2XBH6	Myosin-binding protein H-like	-1.0957	down	7.72E-03
A0A3Q2Y4L3	Metalloproteinase inhibitor 2-like	-1.2898	down	1.29E-03
A0A3Q2Y4J4	ZP domain-containing protein	-1.0157	down	2.92E-04
A0A3Q2XLV2	Caspase-3	-1.4112	down	5.43E-03
A0A3Q2XP65	Aldehyde dehydrogenase 8 family member A1	-1.2136	down	2.02E-03
A0A3Q2YIC3	Fatty acid synthase	-1.5955	down	9.46E-04
A0A067YEJ9	NADH-ubiquinone oxidoreductase chain 1	-1.0098	down	4.71E-03
A0A3Q2YB11	Beta-crystallin A2	-1.603	down	4.77E-02
A0A3Q2XF46	Lipoxygenase domain-containing protein	-1.1247	down	2.81E-02
A0A3Q2YLU8	ATP synthase subunit e, mitochondrial	-1.1639	down	1.06E-03
A0A3Q2XWU4	Beta-crystallin B3	-2.244	down	3.75E-02
A0A3Q3DKY9	Uncharacterized protein	-1.1348	down	1.67E-02
A0A3Q2XSN9	Scavenger receptor class A member 3	-2.4884	down	7.28E-04
A0A3Q2Z3G7	Myosin light chain 7	-1.1555	down	3.93E-03
A0A3Q3E680	Cytochrome c oxidase subunit NDUFA4	-1.6038	down	8.45E-03
A0A3Q2ZCZ7	Pipecolic acid and sarcosine oxidase	-1.3037	down	1.15E-04
A0A3Q3DHT6	Troponin T2d, cardiac	-1.0954	down	2.68E-03
A0A3Q2YMU	Protein-cysteine N-palmitoyltransferase	-1.0746	down	2.45E-02

2	HHAT-like protein			
A0A3Q2YRA3	Annexin	-1.1877	down	4.66E-03
A0A3Q2ZBG3	long-chain-fatty-acid--CoA ligase	-1.2409	down	5.08E-04
A0A3Q2ZDC3	Alpha-2-HS-glycoprotein-like	-1.2402	down	2.89E-03
A0A1Y1BZT8	C-type lectin	-5.245	down	5.72E-5
A0A3Q2YIG1	Equilibrative nucleoside transporter 1-like	-1.189	down	4.61E-03
A0A3Q2ZB40	Beta-crystallin A4	-2.4892	down	4.36E-02
A0A3Q2ZNZ7	Beta-crystallin B1	-2.195	down	3.71E-02
A0A3Q2XMG2	Transferrin receptor protein 1	-1.7447	down	4.18E-04
A0A3Q2Z1T0	Ureidopropionase, beta	-1.061	down	6.04E-03
A0A3Q3DWU2	Beta-crystallin B1-like	-2.6385	down	1.65E-02
A0A3Q2YCJ1	Atypical kinase COQ8A, mitochondrial	-1.1507	down	9.98E-04
A0A3Q3DY25	Protein-glutamine gamma-glutamyltransferase 2-like	-1.2997	down	2.94E-04
A0A3Q2Z2F0	Cytochrome c oxidase subunit 7A-related protein, mitochondrial	-1.1269	down	5.19E-03
A0A3Q3E659	Protein NipSnap homolog 3A-like	-2.5096	down	9.50E-04
A0A3Q2XA34	E3 ubiquitin-protein ligase RNF128-like	-1.3368	down	3.07E-02
A0A3Q3D3K3	Protein S100-A13-like	-1.266	down	3.57E-02
A0A3Q2XLP0	15-hydroxyprostaglandin dehydrogenase	-1.0589	down	1.34E-02
A0A3Q2YNC9	Gamma-crystallin M3-like	-3.028	down	9.32E-03
A0A3Q2Z8H5	Glycerate kinase	-1.0139	down	3.39E-02
A0A3Q2Z7H2	Cytochrome c oxidase subunit	-1.3485	down	6.89E-04
A0A3Q2Y6V3	Cytochrome P450 2K1-like	-1.4647	down	6.54E-03
A0A3Q2XNU0	L-threonine 3-dehydrogenase, mitochondrial-like	-1.0634	down	1.704E-6
A0A3Q3DAU4	D-amino-acid oxidase	-1.1078	down	1.33E-03
A0A3Q2Z167	Protein FAM162B-like	-1.0801	down	3.49E-02
A0A3Q2YSG3	Signal peptide peptidase-like 2A	-1.1966	down	1.30E-03
A0A3Q2XRX1	Matrix metalloproteinase-9	-2.1877	down	8.34E-03
A0A3Q2XQZ2	Bridging integrator 2-like	-1.0072	down	1.27E-02
A0A3Q2XXY8	Long-chain-fatty-acid--CoA ligase	-1.3677	down	6.49E-03
A0A3Q2YM13	Transmembrane 4 L6 family member 5-like	-1.0713	down	3.71E-03
A0A3Q2XLE8	Zona pellucida protein AX 1	-1.5881	down	1.35E-04
A0A3Q2XZA6	Bile salt export pump-like	-1.304	down	7.24E-5
A0A3Q3D4U7	NADH dehydrogenase [ubiquinone] 1 alpha subcomplex subunit 12	-1.0544	down	5.11E-03
A0A3Q2YMQ	Zona pellucida sperm-binding protein 4	-1.7103	down	6.16E-04

7				
A0A3Q2YNX8	L-rhamnose-binding lectin SML-like	-2.0825	down	3.42E-03
A0A3Q2Y044	Sequestosome 1	-1.3514	down	2.83E-02
A0A3Q2YZC8	Solute carrier family 2 member 2	-2.1629	down	9.519E-5
A0A3Q2XJ36	Mitochondrial brown fat uncoupling protein 1	-1.3273	down	5.32E-03
A0A3Q2Z1Q8	Natriuretic peptides A-like	-2.0368	down	8.06E-04
A0A3Q2Z365	24-dehydrocholesterol reductase	-1.586	down	1.27E-03
A0A3Q2XH73	Ig-like domain-containing protein	-1.0628	down	1.09E-02
A0A3Q2Y098	Glycine cleavage system P protein	-1.1646	down	8.25E-04
A0A3Q2Z8D1	glutaminase	-1.4755	down	1.66E-03
A0A3Q2X959	Heat shock protein family B (small) member 8	-1.0271	down	5.96E-03
A0A3Q2Y0C6	Kazal-like domain-containing protein	-1.6593	down	1.13E-02
A0A3Q2YNP8	Zgc:101858	-1.41	down	1.65E-03
A0A3Q2YDM6	Proline dehydrogenase	-1.1066	down	1.25E-02
A0A3Q3DUU1	Cytochrome b-245 heavy chain	-1.162	down	2.35E-03
A0A3Q2Z539	Glutathione S-transferase kappa	-1.287	down	3.08E-02
A0A3Q2XYC3	methenyltetrahydrofolate cyclohydrolase	-2.2009	down	7.26E-04
A0A3Q2Y6V1	Transporter	-2.6841	down	6.22E-04
A0A3Q2Z6J8	Lens fiber major intrinsic protein	-2.7563	down	3.58E-02
A0A3Q2Y9Q9	Inositol 1,4,5-trisphosphate/ryanodine receptor domain-containing protein	-1.2831	down	4.93E-02
A0A3Q2YGD1	Cytochrome P450 2F2-like	-1.4836	down	2.32E-04
A0A3Q2YTG4	Signal peptidase complex subunit 1	-2.1578	down	1.38E-02
A0A3Q2YAF9	Translocator protein	-1.5374	down	2.09E-03
A0A3Q2XV06	Tubulin--tyrosine ligase	-1.2311	down	1.88E-02
A0A3Q2XXZ6	UDP-glucuronosyltransferase	-1.6795	down	7.27E-04
A0A3Q3DHW3	Muscular LMNA interacting protein	-1.0067	down	7.32E-03
A0A3Q2YRE5	Growth hormone inducible transmembrane protein	-1.707	down	2.23E-03
A0A3Q2ZN97	Zonadhesin-like	-1.4197	down	5.11E-03
A0A3Q2XY11	HECT-type E3 ubiquitin transferase	-1.1367	down	1.67E-02
A0A3Q3DEI1	Allograft inflammatory factor 1-like	-1.7696	down	2.12E-04
A0A3Q2X8F6	ATP binding cassette subfamily F member 2	-1.2263	down	5.29E-03
A0A3Q2XSL4	Uncharacterized protein	-1.3978	down	2.55E-02
A0A3Q2YW86	Solute carrier family 38 member 4	-1.4739	down	1.12E-03
A0A3Q2YY89	Galectin	-1.1646	down	3.59E-02
A0A3Q2YNT3	ATP binding cassette subfamily D member 3	-1.7013	down	1.60E-03

A0A3Q2Z788	glutaminase	-1.3748	down	1.44E-03
A0A3Q2XXZ2	Zgc:64106	-1.5636	down	1.33E-04
A0A3Q2XB66	Dehydrogenase/reductase 7C	-1.3408	down	2.23E-02
A0A3Q2ZFS9	Tapasin-related protein-like	-1.1367	down	4.90E-03
A0A3Q2Y5M4	Flavin-containing monooxygenase	-1.4173	down	4.27E-02
A0A3Q2ZDV1	Abhydrolase domain containing 6	-1.4787	down	5.28E-04
A0A3Q3DPQ4	Twinfilin actin binding protein 2	-1.3335	down	3.20E-02
A0A3Q3DE30	Claudin	-1.6122	down	1.25E-02
A0A3Q2YAC7	Cytochrome P450 2F3-like	-1.7889	down	8.271E-5
A0A3Q2XQ63	Myosin-binding protein C, cardiac-type	-1.3126	down	3.86E-02
A0A3Q3D6P0	Ceramide synthase 2-like	-1.2179	down	5.58E-03
A0A3Q2Z5S3	protein-serine/threonine phosphatase	-1.2152	down	2.74E-03
A0A3Q2Y8V8	Flavin-containing monooxygenase	-1.4775	down	7.78E-04
A0A3Q3DSE2	Y-box-binding protein 2-A-like	-1.1053	down	1.17E-02
A0A3Q2Y1Z1	Sarcalumenin	-1.1714	down	2.40E-02
A0A3Q2XCA4	Solute carrier organic anion transporter family member	-1.2324	down	6.31E-03
A0A3Q2Y8L6	long-chain-fatty-acid--CoA ligase	-1.4596	down	9.043E-5
A0A3Q2XH97	Villin-like	-2.2997	down	1.57E-02
A0A3Q3ECS7	Calpain 9	-1.1613	down	1.47E-02
A0A3Q3D8C2	Dehydrogenase/reductase SDR family member 7C-B-like	-1.4301	down	6.66E-04
A0A3Q2XL87	Growth/differentiation factor 8	-1.2334	down	8.32E-03
A0A3Q2ZGW5	peptide-methionine (S)-S-oxide reductase	-1.189	down	3.10E-04
A0A3Q3DJ61	Trafficking protein particle complex subunit 13	-1.2382	down	3.90E-02
A0A3Q2XVA3	P43 5S RNA-binding protein-like	-12.5112	down	1.38E-03
A0A3Q2XMR9	acetyl-CoA carboxylase	-1.1591	down	5.75E-03
A0A3Q2Y0V7	Sodium-coupled neutral amino acid transporter 3-like	-1.2447	down	7.63E-03
A0A3Q3DMS5	Voltage-dependent L-type calcium channel subunit alpha	-1.6371	down	2.90E-03
A0A3Q2YWH8	Vacuole membrane protein 1	-1.6148	down	4.92E-04
A0A3Q2YSV9	Arachidonate 5-lipoxygenase activating protein	-1.0559	down	3.31E-02
A0A3Q2XMB8	Anion exchange protein	-1.1001	down	3.65E-02
A0A3Q2Y893	P-type domain-containing protein	-1.0487	down	1.91E-02
A0A3Q3DC39	Beta-crystallin A1-like	-2.6897	down	5.81E-03
A0A3Q2XSM5	Solute carrier family 43 member 1	-1.3058	down	3.57E-02

A0A3Q2XAA9	Sterol 26-hydroxylase, mitochondrial-like	-1.3434	down	4.07E-04
A0A3Q2ZNF4	Inositol 1,4,5-trisphosphate receptor	-1.3223	down	1.13E-02
A0A3Q2YAD8	Natural resistance-associated macrophage protein 2-like	-1.3937	down	9.05E-03
A0A3Q2Z7U0	Guanine nucleotide-binding protein subunit beta-5-like	-1.5847	down	9.70E-03
A0A3Q2YG14	Nucleotide binding protein like	-1.0268	down	2.10E-02
A0A3Q2Y3U5	ATP binding cassette subfamily A member 1	-1.0565	down	1.49E-02
A0A3Q2YSR4	Apoptosis regulator BAX-like	-1.5027	down	3.04E-02
A0A3Q2XVJ2	Protein phosphatase 1 regulatory subunit 1B	-1.6403	down	3.94E-03
A0A3Q2YIT7	NADH-cytochrome b5 reductase	-1.6818	down	3.37E-03
A0A3Q3DHU6	Caldesmon 1b	-1.3681	down	1.57E-02
A0A3Q2Z2B8	stearoyl-CoA 9-desaturase	-2.1297	down	6.55E-04
A0A3Q2Z3K1	Solute carrier family 38 member 3b	-1.3662	down	2.99E-03
A0A3Q2YVZ5	Cystatin-B-like	-1.0327	down	3.11E-03
A0A3Q2YAD6	Pinin	-1.0309	down	1.53E-02
A0A3Q2XU45	Ovochymase-2-like	-1.5146	down	5.04E-03
A0A3Q3D837	Amino acid transporter	-1.3252	down	1.19E-02
A0A3Q3D2M5	Normal mucosa of esophagus-specific gene 1 protein	-1.2399	down	2.38E-03
A0A3Q2Z6A3	Carbonic anhydrase	-1.4929	down	1.97E-02
A0A3Q3DXD3	Zgc:123305	-1.0457	down	6.83E-03
A0A3Q2YAJ0	RNA binding protein, mRNA processing factor 2	-1.3397	down	2.71E-02
A0A3Q3D7H9	Extracellular serine/threonine protein kinase FAM20C-like	-1.1247	down	2.13E-03
A0A3Q3D959	Trypsinogen-like protein 3	-2.3126	down	2.35E-04
A0A3Q2YQC9	Claudin	-12.2043	down	2.21E-04
A0A3Q3DZR8	Glutaminyl-peptide cyclotransferase	-1.3437	down	3.23E-02
A0A3Q2YQQ1	Epidermal growth factor receptor kinase substrate 8-like protein 3	-1.0514	down	6.80E-03
A0A3Q2YZ16	Long-chain-fatty-acid--CoA ligase	-1.228	down	1.29E-02
A0A3Q2Z9V7	Fatty acid amide hydrolase 2	-1.3119	down	1.29E-02
A0A3Q3E3K4	Peroxisomal membrane protein PEX14	-1.1838	down	1.37E-02
A0A3Q2YB16	Charged multivesicular body protein 5	-1.0616	down	2.36E-02
A0A3Q2YRN1	Major facilitator superfamily domain containing 2A	-2.3169	down	4.48E-04
A0A3Q2YL11	Purine nucleoside phosphorylase	-1.1137	down	6.91E-03
A0A3Q2YX56	Solute carrier family 22 member 16	-1.7554	down	3.59E-03
A0A3Q3DK69	Hematopoietic lineage cell-specific	-1.2358	down	1.38E-02

	protein-like			
A0A3Q2YCN3	Nuclear receptor interacting protein 2	-1.0908	down	2.15E-02
A0A3Q3D1U3	Mitochondrial coenzyme A transporter SLC25A42	-1.0472	down	3.05E-02
A0A3Q2XQJ4	Aconitate decarboxylase 1	-1.0529	down	2.63E-02
A0A3Q2XYK2	CL004 protein	-1.1175	down	2.78E-02
A0A3Q2XMK2	UDP-N-acetylglucosamine--dolichyl-phosphate N-acetylglucosaminephosphotransferase	-1.3489	down	8.52E-04
A0A3Q3D5B7	Copper transport protein	-2.3372	down	1.52E-03
A0A3Q2Y4C4	protein-serine/threonine phosphatase	-1.4185	down	6.28E-03
A0A3Q2XK17	Dpy-19 like C-mannosyltransferase 1	-1.4635	down	3.36E-02
A0A3Q2XIE1	Carcinoembryonic antigen-related cell adhesion molecule 21-like	-1.3004	down	3.94E-03
A0A3Q2ZJX2	Rhodopsin	-1.0502	down	3.47E-02
A0A3Q2Y2K3	long-chain-fatty-acid--CoA ligase	-1.1272	down	2.79E-03
A0A3Q3DYB4	Glycogen [starch] synthase	-1.5249	down	2.10E-02
A0A3Q2Z506	long-chain-fatty-acid--CoA ligase	-1.0289	down	2.52E-02
A0A3Q2XWS6	Glucose-6-phosphatase catalytic subunit 1	-1.1035	down	2.70E-02
A0A3Q2XLS9	Farnesyl pyrophosphate synthase	-1.4628	down	1.03E-02
A0A3Q3ECD8	Lysophospholipid acyltransferase 7	-1.3292	down	2.49E-03
A0A3Q2Y0W3	Fatty acyl-CoA reductase	-11.6932	down	8.20E-04
A0A3Q2Z875	Leucine rich repeat containing 39	-11.6737	down	1.21E-02
A0A3Q2XLQ5	Uncharacterized LOC109520470	-11.6685	down	5.74E-04
A0A3Q2XU61	non-specific serine/threonine protein kinase	-1.3496	down	6.68E-03
A0A3Q2Y196	Neuronal cell adhesion molecule	-11.6476	down	2.20E-02
A0A3Q2Y081	S100/CaBP-9k-type calcium binding subdomain domain-containing protein	-1.3827	down	2.05E-02
A0A3Q3D5K3	Uncharacterized LOC109520318	-1.1081	down	1.67E-02
A0A3Q2XI94	Sm domain-containing protein	-1.0023	down	3.69E-02
A0A3Q2XHB8	RNA-binding protein 25-like	-1.2183	down	1.29E-02
A0A3Q3D9S9	Myosin light chain 1, skeletal muscle isoform	-11.535	down	9.60E-04
A0A3Q2XAV2	General transcription factor IIF subunit 2	-2.0565	down	2.51E-02
A0A3Q2YC05	RAB20, member RAS oncogene family	-11.4365	down	1.67E-02

A0A3Q2ZLR1	Heme oxygenase	-1.3692	down	1.41E-02
A0A3Q3DDX0	Growth hormone receptor	-1.1649	down	4.43E-02
A0A3Q2XRB9	Activating signal cointegrator 1 complex subunit 2	-1.699	down	4.95E-02
A0A3Q2YM45	Phosphoinositide-3-kinase adaptor protein 1	-1.5105	down	1.75E-02
A0A3Q3DVU3	Proteolipid protein 2	-1.923	down	3.00E-02
A0A3Q2XXC8	DNA replication licensing factor MCM6	-1.6855	down	4.44E-02
A0A3Q2XI27	C-type lectin domain-containing protein	-11.322	down	1.435E-5
A0A3Q2X8B2	MAGUK p55 subfamily member 5-A-like	-11.3044	down	4.12E-03
A0A3Q2Z8L1	sn-1-specific diacylglycerol lipase	-11.2744	down	2.916E-5
A0A3Q2XRE6	ER membrane protein complex subunit 6	-11.2311	down	2.06E-02
A0A3Q3E0A9	Angiotensin II receptor associated protein	-11.2187	down	7.53E-04
A0A3Q2XUQ1	G kinase anchoring protein 1	-3.2557	down	1.82E-02
A0A3Q3DGH3	4'-phosphopantetheine phosphatase	-1.5166	down	6.22E-03
A0A3Q2Z286	Glutathione S-transferase	-1.6657	down	3.42E-02
A0A3Q2XLF5	Toll like receptor 3	-1.4639	down	7.31E-03
A0A3Q2Y8K6	Regulator of G-protein signaling 6-like	-11.1149	down	4.09E-04
A0A3Q2YD92	Thyroid hormone receptor interactor 11	-11.06	down	1.56E-04
A0A3Q3DGI2	Actin binding Rho activating protein b	-11.0508	down	8.82E-03
A0A3Q2YVW9	Catenin beta-1	-1.3423	down	4.95E-02
A0A3Q3DY39	Plasmolipin	-10.9039	down	3.69E-02
A0A3Q2XP29	NADH dehydrogenase [ubiquinone] 1 beta subcomplex subunit 2, mitochondrial	-10.8711	down	1.86E-02
A0A3Q3DDA7	Nucleolar GTP-binding protein 1	-1.018	down	1.63E-02
A0A3Q2XQZ9	Protein-serine/threonine kinase	-10.7977	down	2.73E-04
A0A3Q2Z6L0	Elongation of very long chain fatty acids protein 6	-2.4548	down	3.129E-5
A0A3Q3DFE1	Peroxisomal biogenesis factor 19	-10.6182	down	2.08E-03
A0A3Q2YGB8	Lengsin	-10.5453	down	2.40E-02

A0A3Q2XNI7	[histone H3]-lysine(4) N-trimethyltransferase	-10.5354	down	6.40E-04
A0A3Q2YV49	Transmembrane protein 208	-10.4919	down	2.809E-6
A0A3Q2ZIF6	Low-density lipoprotein receptor-like	-10.4593	down	3.31E-03
A0A3Q2YE87	Ig-like domain-containing protein	-10.4212	down	6.91E-03
A0A3Q2XD84	Extracellular matrix protein 1-like	-10.421	down	4.06E-02
A0A3Q2Y8C8	Glucose-6-phosphatase	-10.3523	down	6.15E-03
A0A3Q3DTQ0	Zona pellucida sperm-binding protein 3	-10.3311	down	1.15E-02
A0A3Q2YHQ7	Mitochondrial import inner membrane translocase subunit TIM17	-10.283	down	3.16E-04
A0A3Q2XZ48	Phospholipid-transporting ATPase	-10.1986	down	1.74E-02
A0A3Q2XAI1	Complex III assembly factor LYRM7	-10.1894	down	2.308E-7
A0A3Q2YBL7	E3 ubiquitin-protein ligase	-10.0973	down	1.56E-04
A0A3Q2XCR2	C-C motif chemokine 25-like	-10.0163	down	4.803E-8
A0A3Q2Y707	Polypeptide N-acetylgalactosaminyltransferase	-9.9245	down	2.20E-02
A0A3Q2YDD5	Solute carrier family 30 member 6	-9.8804	down	1.91E-02
A0A3Q2XAQ0	Midasin AAA ATPase 1	-9.8601	down	1.85E-02
A0A3Q3DYP3	Triple QxxK/R motif-containing protein	-9.7554	down	1.78E-03
A0A3Q2Y0V4	AKT1 substrate 1	-9.7442	down	3.29E-02
A0A3Q2Y2R0	Polypyrimidine tract-binding protein 2-like	-9.6932	down	5.88E-03
A0A3Q2XB02	RNA binding motif protein 26	-9.6706	down	6.27E-03
A0A3Q2Z7K2	Anthrax toxin receptor	-9.6255	down	1.44E-03
A0A3Q2XYB3	Protein-serine/threonine kinase	-9.5393	down	6.48E-04
A0A3Q2Z602	J domain-containing protein	-9.4629	down	1.21E-03
A0A3Q2XYE1	Tumor protein p53 inducible nuclear protein 2	-9.3827	down	2.81E-03
A0A3Q2YAE5	Zyg-11 homolog (C. elegans)	-9.2609	down	3.85E-03
A0A3Q2YL04	VRK serine/threonine kinase 3	-9.1347	down	3.22E-02
A0A3Q2Y0P6	Replication factor C subunit 4	-9.0497	down	1.94E-02
A0A3Q2XWZ5	Semaphorin 4D	-8.8695	down	1.88E-02
A0A3Q2Z352	Guanine nucleotide-binding protein	-8.7939	down	3.72E-03

	subunit gamma			
A0A3Q2XY37	dolichyl-phosphate beta-glucosyltransferase	-8.792	down	1.97E-02
A0A3Q3DV64	Solute carrier family 35 member E1	-8.6029	down	1.58E-02

Note: Log_2FC was calculated as log_2(sun-dried/freeze-dried). "Up" indicates higher relative protein abundance in the sun-dried group, and "Down" indicates higher abundance in the freeze-dried group. Differentially abundant proteins were screened at FDR < 0.05. Some protein names are represented by multiple UniProt accessions, which results from paralogous genes and alternative-splicing isoforms in the non-model organism *Hippocampus abdominalis*; different isoforms/paralogs may exhibit opposite abundance changes between the two drying treatments.
